# Gene Network Analysis of Interstitial Macrophages After Treatment with Induced Pluripotent Stem Cells Secretome (iPSC-cm) in the Bleomycin Injured Rat Lung

**DOI:** 10.1007/s12015-017-9790-9

**Published:** 2017-12-18

**Authors:** Luca Tamò, Cedric Simillion, Youssef Hibaoui, Anis Feki, Mathias Gugger, Antje Prasse, Benedikt Jäger, Torsten Goldmann, Thomas Geiser, Amiq Gazdhar

**Affiliations:** 1 Department of Pulmonary Medicine, University Hospital Bern, 3010 Bern, Switzerland; 2 Graduate School for Cellular and Biomedical Sciences, University of Bern, Bern, Switzerland; 3 Department of Biomedical Research, University of Bern, Bern, Switzerland; 4 Interfaculty Bioinformatics Unit, University of Bern, Bern, Switzerland; 5 Department of Gynecology and Obstetrics, University Hospital Geneva, Bern, Switzerland; 6 Department of Gynecology and Obstetrics, Cantonal Hospital Fribourg, Fribourg, Switzerland; 7 Promed Laboratories Fribourg, Fribourg, Switzerland; 8 Hannover Medical School, Clinic for Pneumology, Hanover, Germany; 9 Fraunhofer Institute for Toxicology and Experimental Medicine, Hanover, Germany; 10 Pathology of the University Hospital of Lübeck and the Leibniz Research Center Borstel, Borstel, Germany; 11 Airway Research Center North (ARCN), Member of the German Center for Lung Research (DZL), Groβhansdorf, Germany

**Keywords:** Lung fibrosis, Macrophages, Induced pluripotent stem cells, Stem cell secretome

## Abstract

Idiopathic pulmonary fibrosis (IPF) is a complex disease involving various cell types. Macrophages are essential in maintenance of physiological homeostasis, wound repair and fibrosis in the lung. Macrophages play a crucial role in repair and remodeling by altering their phenotype and secretory pattern in response to injury. The secretome of induced pluripotent stem cells (iPSC-cm) attenuates injury and fibrosis in bleomycin injured rat lungs. In the current study, we evaluate the effect of iPSC-cm on gene expression and phenotype of interstitial macrophage in bleomycin injured rat lungs *in vivo*. iPSC-cm was intratracheally instilled 7 days after bleomycin induced lung injury and assessed 7 days later and single cell isolation was performed. Macrophages were FACS sorted and microarray analysis was performed. We characterized changes in the rat lung interstitial macrophages using transcriptional profiling. iPSC-cm reduced the total collagen content of the lung and reduced different macrophage populations. Gene set enrichment analysis revealed involvement of three essential pathways (a) immune modulation, (b) branching morphogenesis and (c) canonical Wnt signaling. This study demonstrates that iPSC-cm reduces fibrosis in bleomycin injured rat lung by partially altering the macrophages and regulating their gene expression.

**Electronic supplementary material:**

The online version of this article (10.1007/s12015-017-9790-9) contains supplementary material, which is available to authorized users.

## Introduction

Idiopathic pulmonary fibrosis (IPF) is the most common form of idiopathic interstitial pneumonias and is characterized by progressive loss of alveolar epithelial integrity due to dysregulated wound repair response to repeated alveolar microinjuries [1, 2]. Moreover, recruitment and activation of myofibroblasts and exaggerated deposition of extracellular matrix and collagen results in loss of parenchymal architecture and lung function [3]. IPF is a rapidly progressive disease [4] with median survival of 3 years [5] and is characterized by histopathological pattern of usual interstitial pneumonia [6]. Although the exact etiology of IPF is not known, recent evidence suggest a very complex and dynamic process involving various cell types including macrophages [7].

Macrophages play a very critical role in defense, metabolism and maintenance of homeostasis. Macrophages demonstrate distinguished plasticity in acquiring phenotypes that can either promote or resolve fibroproliferative response to injury. Based on their location, pulmonary macrophages are divided into alveolar macrophages (AMs) residing in airways and interstitial macrophages (IMs) located in lung parenchymal tissue [8]. Historically, the macrophages where described as classically activated or M1 and alternatively activated or M2 macrophages. Recent advances, however, have changed our understanding of macrophage phenotypes, plasticity and their specific role in fibrosis [9]. It is now recommended to classify the macrophages based on their secretions rather than surface marker expression [9]. Macrophages are crucial regulators of fibrosis and are seen in close proximity to myofibroblasts and produce profibrotic mediators. AMs have been shown to secrete matrix metalloproteinases [10] and uptake collagen [11]. However, the role of IMs is not fully known. Based on some murine studies, it is considered that they assume a pro fibrotic phenotype [12]. Therefore, targeting these cells to resolve fibrosis could be a promising novel approach.

Currently there is no cure for IPF. Novel approaches like stem cell based therapies have been successfully tested in animal models over past years, but their safety for clinical application is still under investigation [13]. Generation of induced pluripotent stem cells (iPSC) by reprogramming represents a very promising approach in the field of regenerative medicine [14]. However, the fate of iPSC after cell transplantation is debated [15]. In our opinion, the iPSC secretome (conditioned media) represents advantage over cell transplantation. Its effect has been successfully demonstrated by attenuation of fibrosis in bleomycin injured rat lungs by secretome of induced pluripotent stem cells (iPSC-cm) [16, 17]. However, mechanisms leading to the beneficial effect of iPSC-cm are not fully known. To further understand the antifibrotic mechanisms of iPSC-cm, in the current study we focused on its effect on macrophages. We therefore conducted a microarray experiment to investigate the effect of iPSC-cm on phenotype and gene expression pattern of interstitial macrophages in the bleomycin injured rat lung. We performed Gene Set Enrichment Analysis (GSEA) to detect which pathways are being affected after treatment with iPSC-cm in the macrophages. The most significant pathways were then further investigated using network analysis techniques to find which genes and gene interactions play a crucial role in the observed phenotypic changes.

## Materials and Methods

### Generation of Induced Pluripotent Stem Cells

Human foreskin fibroblasts (line CRL-2429; ATCC, Rockville, MD, USA) were used to generate iPSC as previously described [16]. Cells were maintained in KnockOut™ DMEM supplemented with 20% KnockOut™ Serum Replacement, 2 mmol/l L-Glutamine, 40 µg/ml gentamycin, 100 µmol/l β-mercaptoethanol (Gibco, NY, USA) and 10 ng/ml of human fibroblast growth factor-basic (bFGF; PeproTech, UK). Medium was changed every day to maintain the cells undifferentiated. iPSC colonies were mechanically passaged every 4–5 days as described [16]. For characterization the iPSC colonies were immunostained with Oct ¾ (1:50), NANOG (1:50), Sox-2 (1:50), TRA-1-81 (1:50), TRA-1-60 (1:50) (Santacruz Biotechnologies, USA).

### iPSC-cm Collection

10–12 iPSC colonies (6.5 ± 0.53 × 10^5^) live cells were grown in feeder free condition on plates coated with vitronectin (Stem cell technologies, Canada) one day later the medium was switched to KnockOut™ DMEM prepared as mentioned above, but without serum replacement and bFGF. After 24 h, the supernatant was harvested in falcon tubes and the cellar debris removed via centrifugation (300 g x 5 min). The supernatant was aliquoted and stored at − 80 °C. To confirm pluripotency after culture in KO DMEM media with serum and bFGF the colonies were stained with the pluripotency markers as stated above.

### Animals

Male Fisher F344 rats (240 to 280 g) were obtained from Charles River Laboratories GmbH (Sulzfeld, Germany). Experiments were performed in accordance with the standards of the European Convention of Animal Care. The study protocol was approved by the University of Bern Animal Study Committee.

### Instillation of Bleomycin

At day 0, animals were anesthetized by inhalation of 4% isoflurane in anesthesia chamber and intubated with 14G catheter (Insyte, Spain), and instilled with bleomycin (Baxter, USA) (1.28 U/rat) in a volume of 500 µl.The dosage of bleomycin was based on previously published experiments [18].

### Instillation of iPSC-cm or Control Media

Seven days after instillation of Bleomycin, rats were anesthetized as mentioned above and an intratracheal instillation of either iPSC-cm (n = 5) or control media (Ko media without serum replacement and bFGF (n = 5) was performed in a volume of 500 µl. Additional animals (n = 5) served as normal controls. For time course experiments additional animals were instilled with bleomycin as described and where randomly divided into 2 groups (a) iPSC-cm (b) control media, and animals were sacrificed at 24 h, 48 h 72 h or 7 days after instillation of respective media (n = 3) in each group for each time point.

### Assessment

At day 14 (7 days after iPSC-cm or control media instillation), the animals were anesthetized following the same procedure as above and euthanized with intraperitoneal administration of 50 mg/kg thiopental. The heart–lung block was removed, rinsed with PBS and were either frozen at -80 °C for RNA isolation or fixed in paraformaldehyde for histology, or were directly used for single cell isolation as described below.

### Preparation of Cell Suspension

Resected lungs were mechanically minced in a 10 cm Petri dish (BD) with surgical scissor and incubated in a sterile solution of RPMI 1640 (Gibco,USA) containing 0.1% collagenase I, 0.25% collagenase II (Worthington Biochemical Corporation, NJ, USA) and 2% FBS (Gibco). The Petri dish was incubated at 37 °C and 5% CO_2_ for 90 min, every 15 min the lung undergoing digestion was pipetted up and down to help the cells to disaggregate. The cells suspension was consecutively strained in 100 µm and 40 µm filters (SPL Life Sciences, S.Korea). Cells were then washed with fresh RPMI 1640 and centrifuged at 1680 rpm for 15 min at room temperature. Red blood cell lysis buffer (eBiosciences, USA) was added to the suspensions of cells according to manufacturer instructions to eliminate red blood cells. After lysis, the cells were washed and kept in PBS with 2% FBS at 4 °C for further analysis.

### Antibody Labelling and Flow Cytometry (FACS)

A minimum of 1 × 10^6^ cells from the cell suspension were rinsed with washing buffer (WB) containing PBS, 0.1% BSA (Sigma) and 0.09% NaN_3_ (Sigma). Cells were incubated in 100 µl of washing buffer with the appropriate antibody cocktail for surface markers for 30 min at 4 °C, protected from light. The cells were then permeabilized with PBS containing 2% FCS, 1% EDTA 0.5M pH 8.0, 0.1% saponin and 0.09% NaN3 and further stained with the intracellular marker CD68:AF488 (AbD Serotec,USA).

Antibodies were diluted in WB at concentration of 5 µl/ml AF488 anti-CD68, 3 µl/ml AMCA anti-CD163, 3 µl/ml APC-Cy7 anti-CD206, 3 µl/ml PE anti-CD86 and 3 µl/ml AF647 anti-CD11b.

The same amount of cells was used for isotype and fluorescence minus one (FMO) controls. Compensation was achieved using OneComp eBeads (eBioscence, San Diego, USA) following manufacturer instructions. At least 5 × 10^5^ events were acquired using LSRII (SORP) flow cytometers (BD Biosciences, USA) and data was analyzed using FlowJo software 10 (FlowJo Enterprise, USA).

### Sorting of Macrophages

Lungs of rats were prepared for cell suspension following the procedure mentioned above using a mix of collagenases. The cell suspension was stained with an anti-rat macrophage marker PE (Affymetrix, USA cat no.12–0660) and living cell marker 7-AAD (7-aminoactinomycin D) (BioLegend,USA). Sorting was performed using a FACS Aria III (BD Biosciences, USA). The freshly sorted cells were collected and RNA extraction was performed as described below. For FACS plot please see supplementary figure (Fig S-1).

### Histology and Immunohistochemistry

For immunohistochemistry samples were deparaffinized in series of xylene washes and sequentially re-hydrated following decreasing concentrations of ethanol. For routine histology the slides were stained with hematoxylin and eosin (H&E) staining. For immunohistochemistry the slides were pretreated in sodium citrate buffer (100 mM, pH 7.0) and boiled in a microwave oven for 5 mins. Slides were washed three times with Tris Buffer Saline with 0.1% Tween-20 (TBST). Thereafter, tissue slides were incubated with primary antibodies (CD68,(1:100),CD163,(1:100)(BioRad,USA), CD206 (1:100)(SantaCruz, USA), CD86 (1:100) (BD Bioscience, USA) diluted 1:100 overnight at 4 °C in PBS containing 0.1% Tween-20 and 0.01% triton X-100. Slides were further processed using EnVision™ detection systems (Dako, USA) following the manufacturer’s instructions. The slides were then counterstained using hematoxylin, and mounted. Images were acquired using Leica DM4000D (Leica, AG Germany).

### RNA Isolation

Total RNA was extracted from the freshly sorted cells using the kit NucleoSpin® RNA (MACHEREY–NAGEL, Düren, Germany) according to the manufacturer‘s protocol.The concentration was determined using a NanoDrop ND-1000 spectrophotometer (NanoDrop Technologies, Wilmington, DE). RNA integrity was determined by assessing an aliquot of each RNA sample on an Agilent Bioanalyzer (Agilent Technologies, Palo Alto, CA).

### Microarray

Labeling was performed using the Agilent LowInput QuickAmp Labeling Kit One-Color (5190 − 2305; Agilent Technologies).The SurePrint G3 Rat GE 8 × 60K Kit were used (G4853A, Agilent Technologies). Briefly, first-strand cDNA synthesis was performed using an oligo(dT) 24 primer containing a T7 RNA polymerase promoter site. The cDNA was used as a template to generate Cy3-labeled cRNA that was used for hybridization. After purification and fragmentation, aliquots of each sample were hybridized to Agilent Oligo Microarrays (G2534-60014, Agilent Technologies). After hybridization, each array was sequentially washed and scanned by Agilent microarray scanner. Arrays were individually visually inspected for hybridization defects, and quality control procedures were applied as recommended by the manufacturer of the arrays. For array readout, Agilent Feature Extraction 9.5.3 Software was used, and microarray data were imported, log2-transformed and quantile normalized using robust multi-array average (RMA), and expression levels were summarized on a transcript level using average gene expression values of the replicated probes, as recently described [19]. Differential gene expression was calculated using the moderated t-test as described by Ritchie et al. [20] and implemented in the R/Bioconductor package limma.

### Gene Set Enrichment Analysis

The results of the differential expression analysis was used to perform gene set enrichment analysis (GSEA) using the SetRank method [21]. The key principle of this algorithm is that it discards gene sets that have initially been flagged as significant, if their significance is only due to the overlap with another gene set. It calculates the p-value of a gene set using the ranking of its genes in the ordered list of p-values as calculated by limma and therefore does not require the input gene list to be divided into significant and non-significant genes using a p-value cutoff. The following databases were searched for significant gene sets: BIOCYC [22], Gene Ontology (GO) [23], ITFP [24], KEGG [25], PhosphoSitePlus [26], REACTOME [27], and WikiPathways [28].

### Network Analysis

For each of the significant gene sets returned by SetRank, all protein–protein interactions were retrieved from the STRING [29] database and visualized in Cytoscape [30]. For each edge in the resulting networks, a cost score was calculated as the logarithm of the geometric mean of the adjusted p-value of the interacting genes. This score was then taken into account to calculate for every node and every edge a betweenness value using the igraph package for R [31] Betweenness centrality quantifies the number of times the shortest paths between any two nodes passes through a given node. This betweenness was used to evaluate the importance of individual genes in a network, as described in 21.

### Ashcroft Scoring and Sircol Assay for Assessment of Collagen

Routine hematoxylin and eosin staining was performed with formalin-fixed tissue sections. To evaluate the extent of pulmonary fibrosis, the scoring system of Ashcroft [32] was used by a trained pathologist as previously reported [18]. For Sircol collagen assay Lung tissue was minced and weighted and amount of acid soluble collagen was assessed using Sircol collagen assay (Biocolor Ltd, County Antrim UK) according to the manufacturer’s instructions. The wet weight was measured and lungs were snap frozen. The frozen lung was homogenized in 1x PBS and the homogenate was treated with Sircol dye reagent for 30 min at room temperature. After brief centrifugation the pellet was dissolved in alkali reagent and was measured at 540 nm using Tecan M1000 plate reader (Tecan, AG Switzerland).

### Statistics

The graphs were constructed and the data analyzed using GraphPad Prism software (GraphPad 7, San Diego, CA, USA). Data are shown as mean ± SEM. Statistical analysis was performed using a two tailed unpaired Student’s t-test. Differences with P values ≤ 0.05 were considered significant.

## Results

### Secretome of iPSC Modulates Macrophage Percentage in Vivo

**Fig. 1:**
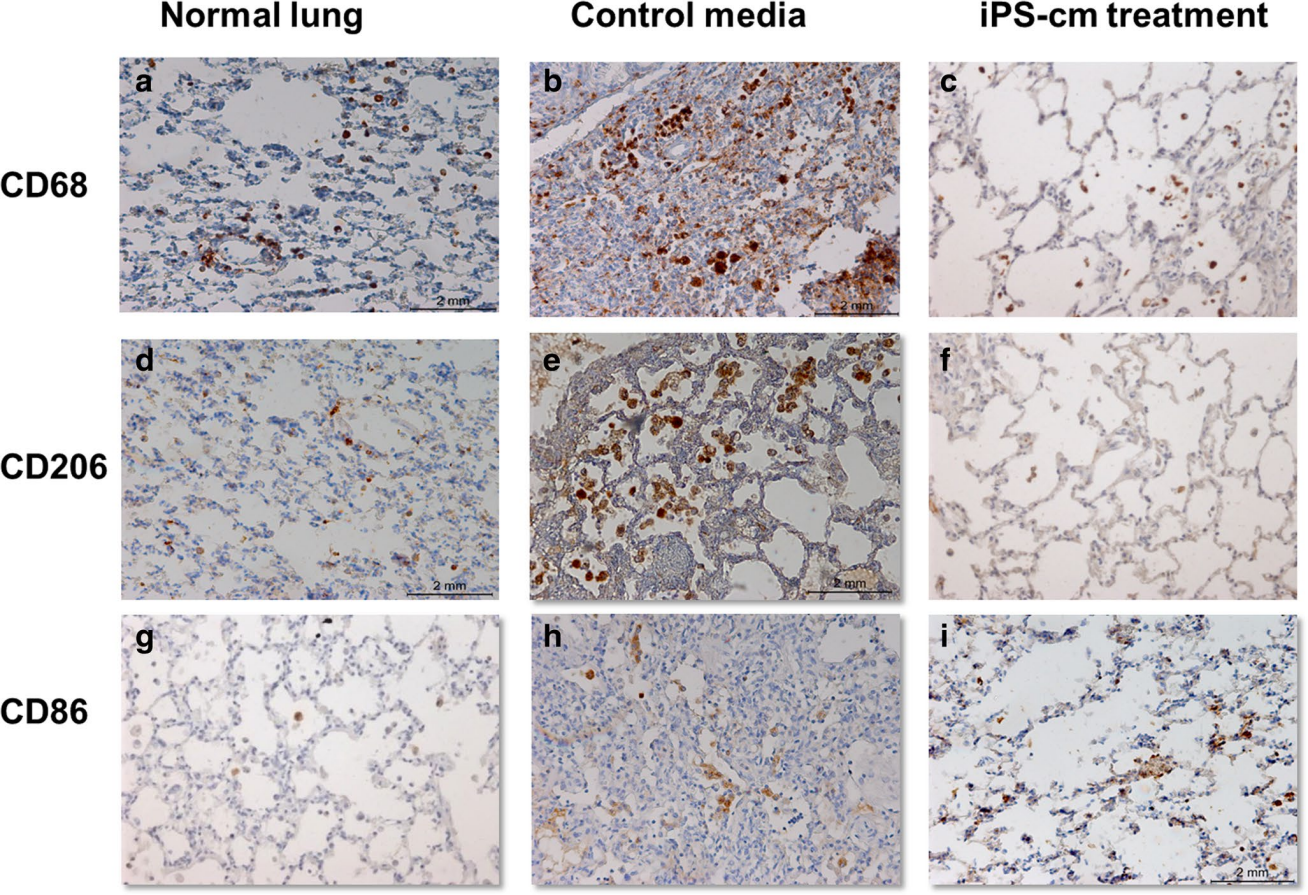
Immunohistochemistry on rat lungs slides from control group show more infiltration of macrophages in the interstitium and in the alveolar space (b). Positive staining for M2 cells (e) and M1 (h) was also found in the lung treated with control media. After iPSC-cm treatment, lung structure was improved and less macrophages are seen in both the interstitium and in the alveolar space (c). Furthermore, M2 (f) and M1 (i) macrophages were also reduced. Panels a,d, and g show the same macrophage markers in normal rat lung

Immunohistochemistry of formalin fixed tissue was performed. Normal lung sections were stained for CD68 (Fig. 1a), CD206 (Fig. 1d), and CD86 (Fig. 1g). Immunostaining revealed increased macrophage staining in bleomycin injured lungs treated with control media CD68 (Fig. 1b), CD206 (Fig. 1e), and CD86 (Fig. 1h). In contrast, macrophages showed reduced staining for CD68 (Fig. 1c), CD206 (Fig. 1f), and CD86 (Fig. 1i) 7 days after treatment with iPSC-cm, compared to animals treated with control media (Fig. 1b and e).

### Percentage of Macrophages is Altered After iPSC-cm Treatment

**Fig. 2:**
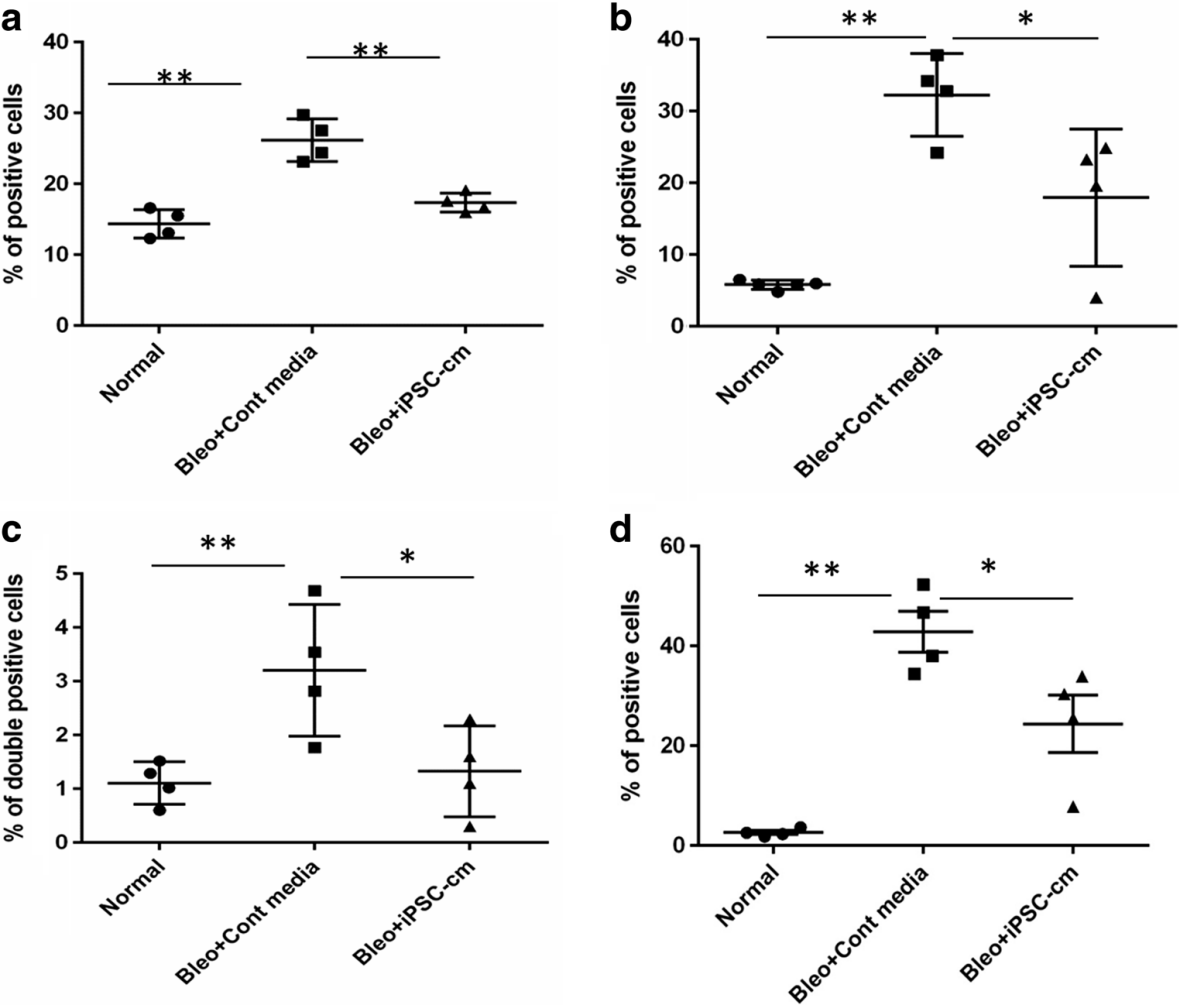
Percentage of total macrophages (CD68^+^) present after single cell isolation (**a**). Percentage of M1 (CD86^+^) (**b**) and M2 (CD206^+^) (**c**) macrophages. Percentage of macrophages positive for both M1 and M2 markers (CD68^+^CD206^+^CD86^+^) (**d**)

Single cells isolated from lung parenchyma were stained for various macrophage markers and FACS analysis was performed. In the normal lung 14.38 ± 1.00% of the total cells stained positive for CD68. In the bleomycin injured animals treated with control media, CD68 positive cells increased to 26.18 ± 1.49%, (p˂0.0006) in the lungs treated with iPSC-cm the number of CD68 positive cells reduced to 17.35 ± 0.66% (p˂0.017)(Fig. 2a). A similar trend was observed with specific markers of M1 and M2 macrophages. In the group treated with control media the level of CD86 (M1 marker) increased to 32.25 ± 2.88% compared to 5.8 ± 0.27% p˂0.0001 in the healthy lung. After iPSC-cm treatment, the number of CD86 positive cells was significantly reduced to 17.95 ± 4.71% p˂0.048 (Fig. 2b).

The percentage of cells positive for both the M2 markers CD163 and CD206 in healthy lung was 1.10 ± 0.19%. The percentage of M2 macrophages increased to 3.20 ± 0.62% in the group treated with control media p˂0.003. However, in the iPSC-cm treated animals the percentage reduced to 1.32 ± 0.42% p˂0.04 (Fig. 2c). Percentage of cells expressing both the markers CD86 and CD206 showed increase in animals treated with control media (42.85 ± 4.07%) compared to healthy lung (2.60 ± 0.39%) p˂0.173, in the iPSC-cm treated animals the percentage of double positive cells dropped to (24.40 ± 5.79%)p˂0.044 (Fig. 2d).

**Fig. 3:**
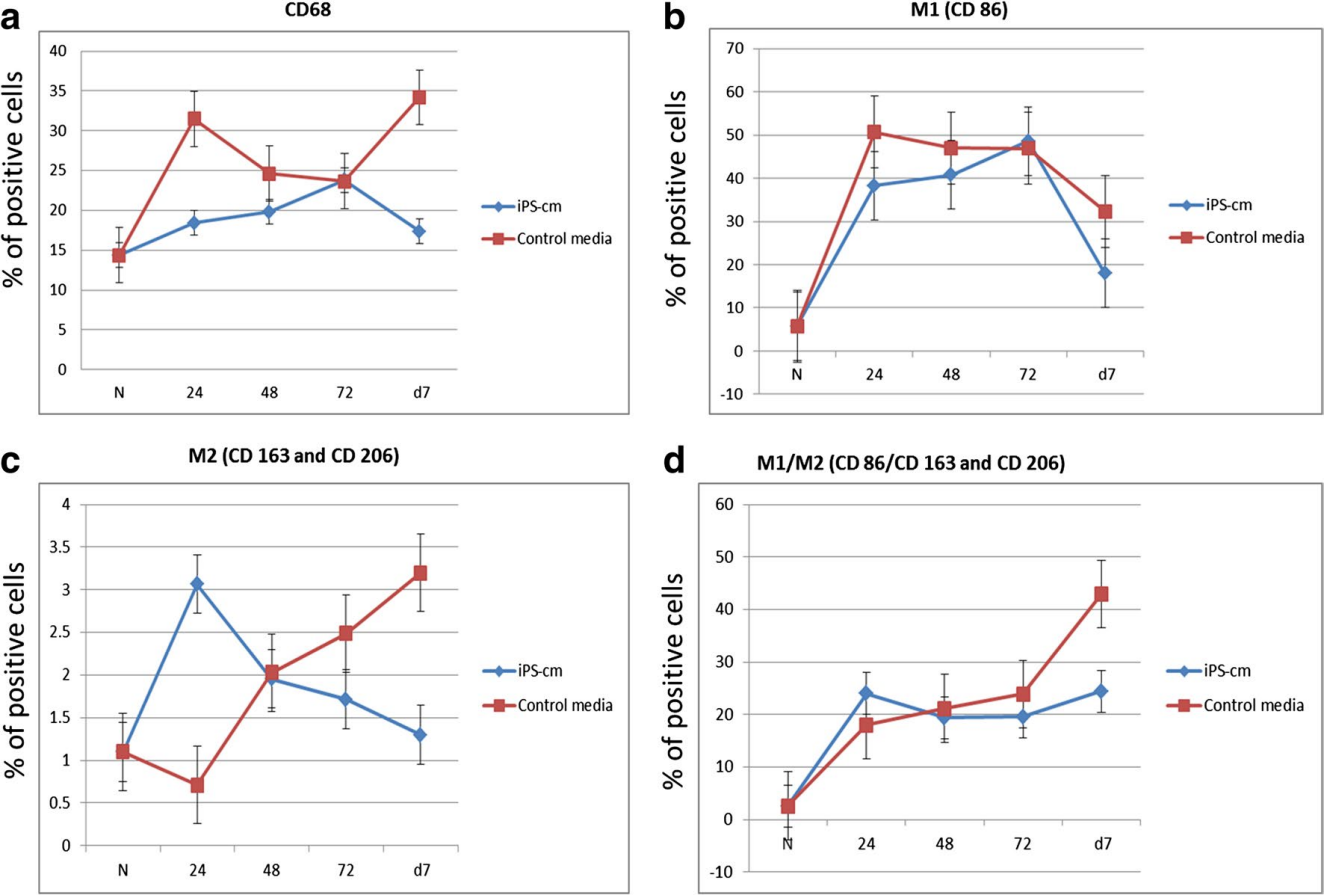
Time dependent effect of iPSC secretome on the percentage of interstitial macrophages (**a**), M1 macrophages (CD86^+^) (**b**), M2 macrophages (CD206^+^) (**c**), M1/M2 macrophages (CD68^+^CD206^+^CD86^+^) (**d**)

Interestingly, the percentage of CD68 positive macrophages decreased over time in the treated group compared to the control group, where increased number of macrophages were observed (Fig. 3a). Moreover, M1 macrophages surged at 24 h in control media and a time dependent decline was observed over 7 days, however in the iPSC-cm treated group M1 macrophages showed sudden increase at 72 h post treatment (Fig. 3b). Interestingly, M2 macrophages surged at 24 h after iPSC-cm treatment, followed by steady decline till day 7 (Fig. 3c). Finally, the double positive macrophages also showed an increase in percentage at 24 h followed by steady decline in treatment group whereas in the control group a time dependent increase was observed over 7 days (Fig. 3d).

### Microarray Analysis

**Fig. 4:**
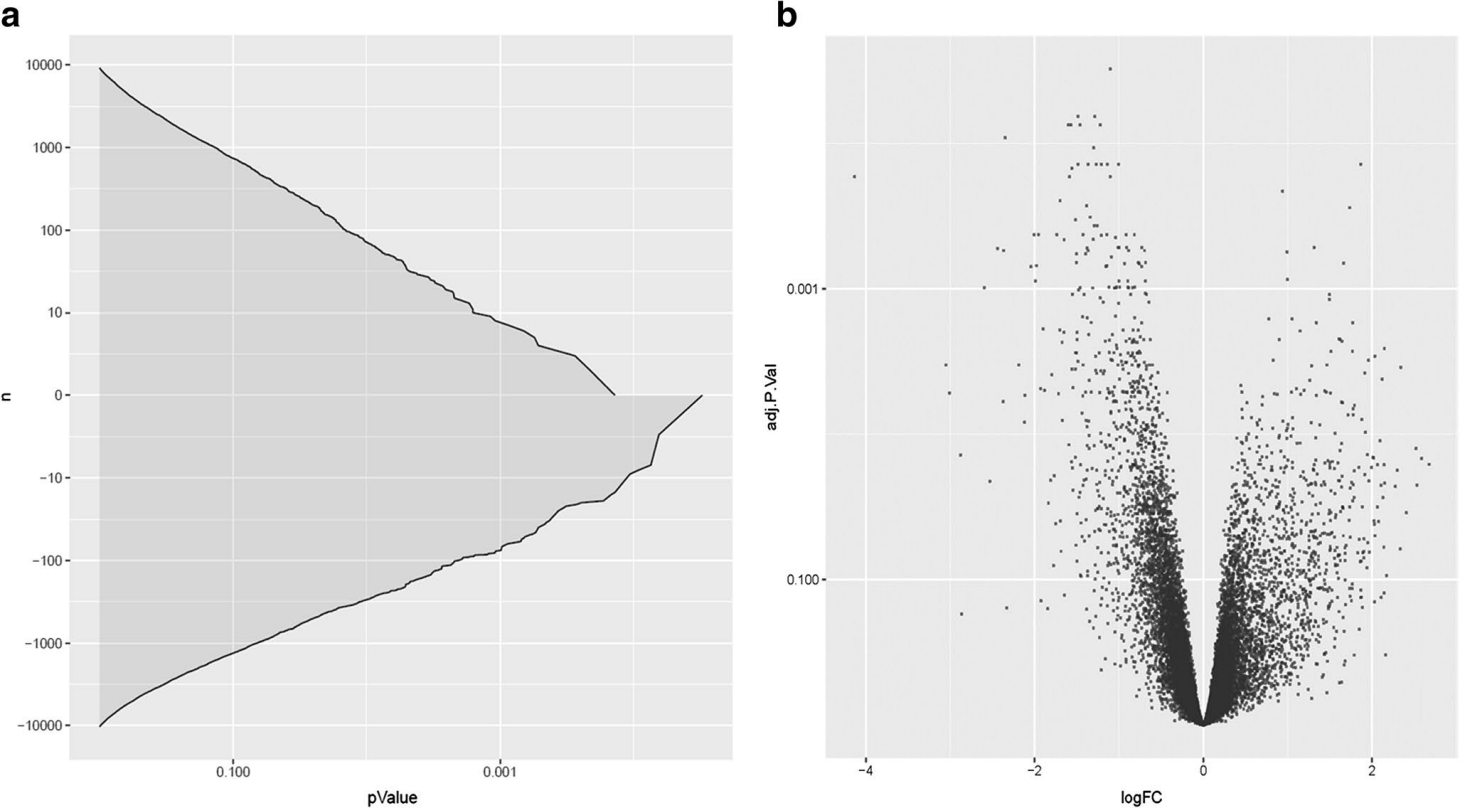
Cumulative adjusted p-value distribution for both up- and downregulated genes in the microarray analysis (**a**). Volcano plot for showing the adjusted p-value as a function of log-fold change for the microarray analysis (**b**)

The microarray analysis revealed upregulation of 384 genes (*p* ≤ 0.05) / 71 genes (*p* ≤ 0.01)/ 7 genes (*p* ≤ 0.001) and downregulation of 831 genes (*p* ≤ 0.05) / 298 genes (*p* ≤ 0.01) / 75 genes (*p* ≤ 0.01) after treatment with the secretome of iPSC (Fig. 4). The raw microarray data are deposited in the ArrayExpress database under accession E-MTAB-5619.

### Pathway Analysis

**Fig. 5:**
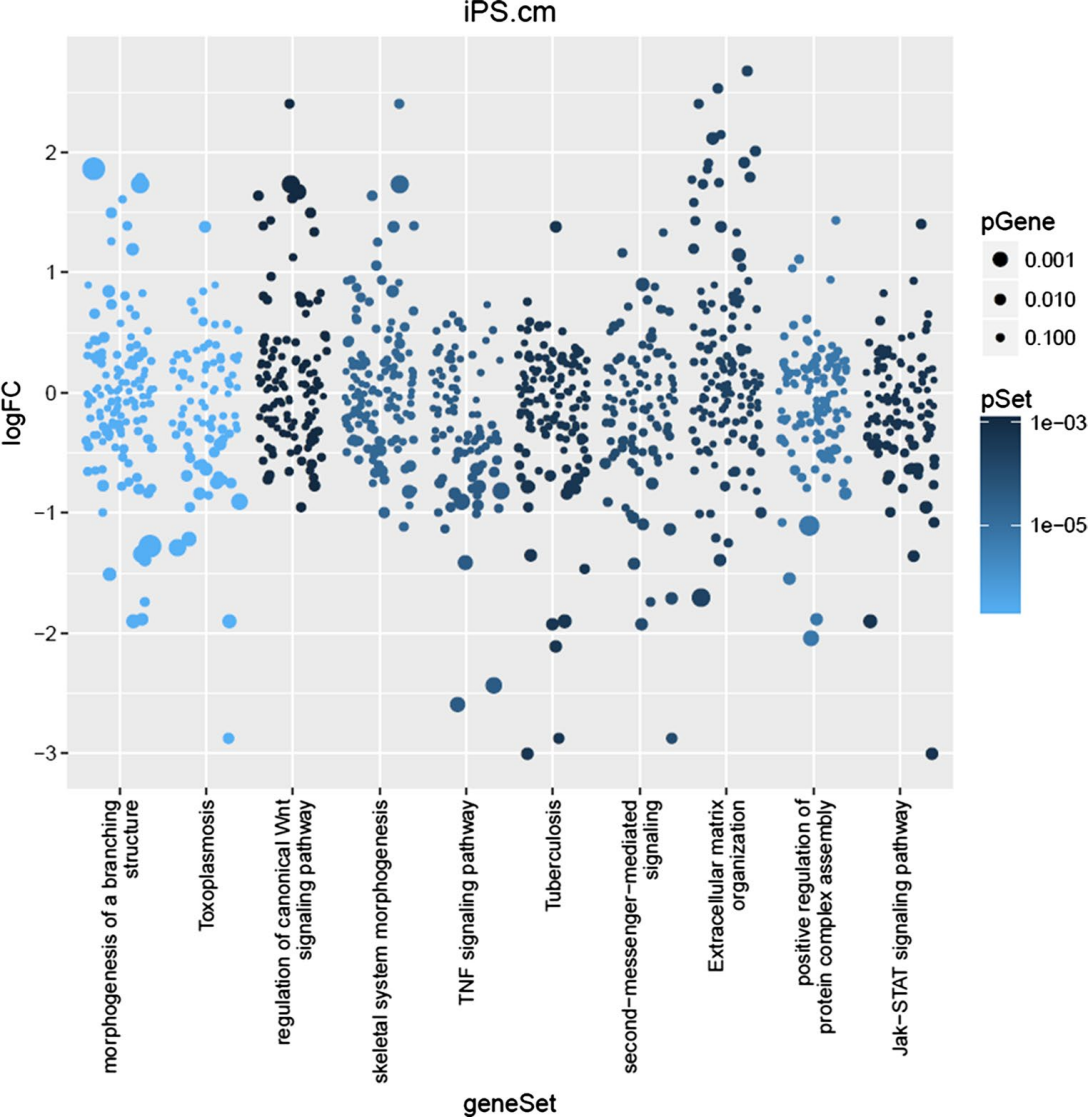
Visualization of the most significant pathways as returned by the SetRank algorithm. Each column represents a pathway. Each dot represents a gene. The size of a dot reflects its significance. The colour of a dot reflects the corrected p-value of the entire pathway. The position on the vertical axis represents the log-fold change value

Gene Set Enrichment analysis using the SetRank algorithm on the differential gene expression returned the following 3 pathways as most significant: “morphogenesis of a branching structure” (GO term GO:0001763), “toxoplasmosis” (KEGG pathway ko05145) and “regulation of canonical WNT signaling pathway” (GO term GO:0060828) (Fig. 5). For each of these pathways, we visualized the known and predicted protein–protein interactions (PPIs), as reported by the STRING database [29]. We then used the betweenness centrality measure to assess the importance of each gene in a given pathway. Betweenness centrality of a given node *N* – i.e. a gene – in a network is defined as the number of all shortest paths connecting any two other nodes *X* and *Y* that pass through *N*.

**Fig. 6:**
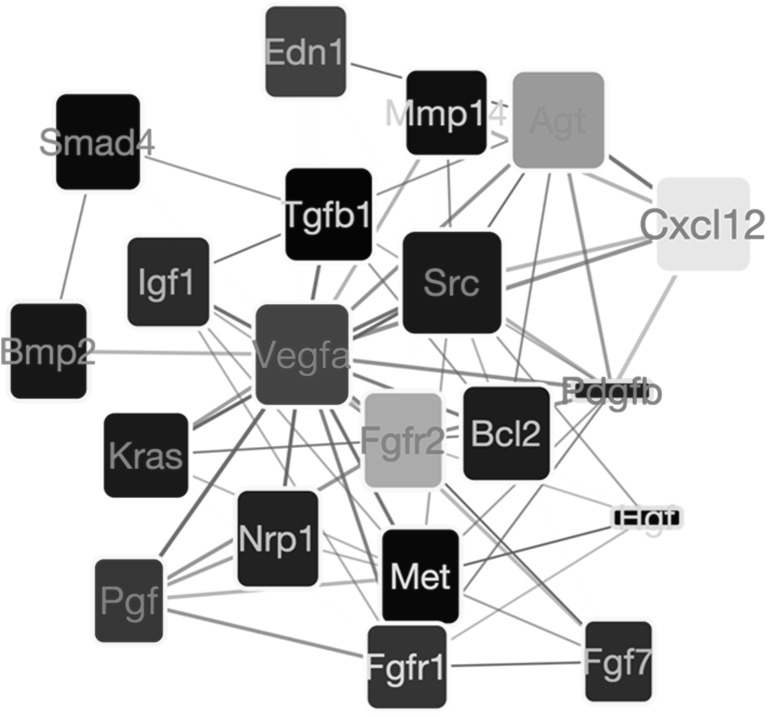
Network showing the most important gene interactions for the “morphogenesis of a branching structure” pathway (GO term GO:0001763). Interactions are retrieved from the STRING database [34]. The fill color of each node reflects the log-fold change of a gene in our microarray experiment with yellow to red indicating up-regulation and blue to cyan indicating down-regulation. The width of a node reflects the betweenness centrality of that node in the entire interactome; the height of a node reflects the betweenness only within this pathway. The node label color indicates how high the gene was ranked in the differential gene expression analysis, going from black to red with increasing significance. Thickness of an edge reflects the evidence strength for an interaction as reported by the STRING database

The most significantly affected pathway is “morphogenesis of a branching structure” (GO term GO:0001763; (Fig. 6). The Src proto-oncogene (Src) and the vascular endothelial growth factor A (Vegfa) have the highest betweenness values in this pathway. Of these two, Vegfa is significantly down-regulated (adjusted p = 0.005) whereas the expression value of Src does not change significantly. This observation suggests that, in this pathway, a dominant role is played by VEGF-a gene. Downregulation of VEGF-a seems to affect several other genes that are responsible for lung remodeling, most notably fibroblast growth factor receptor 2 (Fgfr2), which is up-regulated and Placental Growth Factor (Pgf), a close homolog of VEGF-a, which is also down-regulated. Vegfa especially supports pathological angiogenesis and enhances fibrosis [33].

**Fig. 7:**
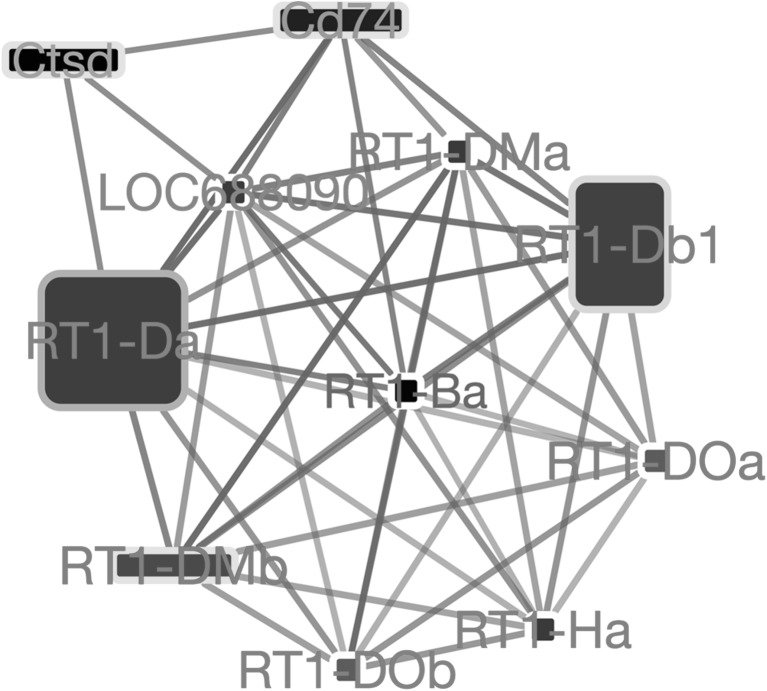
Network showing the most important gene interactions for the “toxoplasmosis” pathway (KEGG pathway ko05145). The gene upregulated are all gene of the MHC family. Interactions are retrieved from the STRING database [34]. The fill color of each node reflects the log-fold change of a gene in our microarray experiment with yellow to red indicating up-regulation and blue to cyan indicating down-regulation. The width of a node reflects the betweenness centrality of that node in the entire interactome; the height of a node reflects the betweenness only within this pathway. The node label color indicates how high the gene was ranked in the differential gene expression analysis, going from black to red with increasing significance. Thickness of an edge reflects the evidence strength for an interaction as reported by the STRING database

The second pathway is referred as “toxoplasmosis” (KEGG pathway ko05145). Network analysis for this pathway revealed a tight cluster of protein–protein interactions, indicative of a protein-complex (Fig. 7). The gene symbols (RT1-DOa, RT1-Da, RT1-Db1, RT1-DMa, RT1-DMb, RT1-Ha, RT1DOb) reveal that this complex is the Major Histocompatibility Complex II (MHC-II). As all nodes in this cluster are interconnected, a betweenness-based analysis is not very useful. As shown by the large label sizes and red label color (see figure legend), almost all genes involved in this complex are slightly but significantly up-regulated. This upregulation indicates an activation of the immune response of the macrophages.

The same group of MHC class II genes is also present in the “Tuberculosis” pathway, which was also detected as significant by our pathway analysis as shown before in (Fig. 5).

The third pathway affected by the secretome is the “regulation of canonical WNT signaling pathway” (GO term GO:0060828; Fig. 8). In this pathway, betweenness values suggest a central role for the Axin 2 (Axin2) gene, which is a potent WNT inhibitor [35]. Axin2 is found to be up-regulated and interacts with Apc2, another suppressor of WNT signalling. It has been showed that M2 macrophages activate WNT signalling pathway in epithelial cells [36]. These observations suggest that treatment with iPSC-cm supresses WNT signalling. Note that Axin2 and its interacting partners are also part of the “skeletal system morphogenesis” pathway (Fig. 9).

**Fig. 8:**
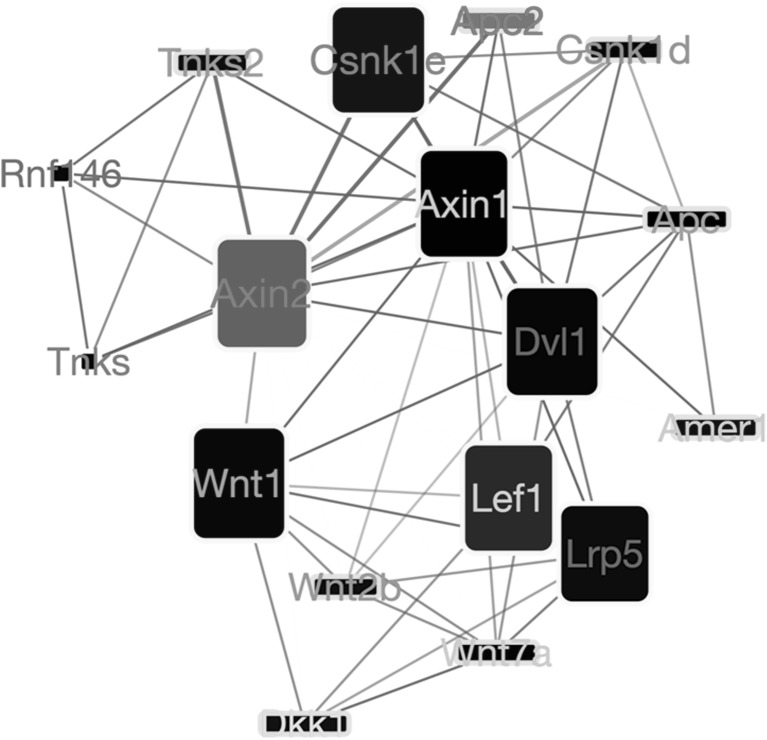
Network showing the most important gene interactions for the “regulation of canonical WNT signaling” (GO term GO:0060828) pathway. Interactions are retrieved from the STRING database [34]. The fill color of each node reflects the log-fold change of a gene in our microarray experiment with yellow to red indicating up-regulation and blue to cyan indicating down-regulation. The width of a node reflects the betweenness centrality of that node in the entire interactome; the height of a node reflects the betweenness only within this pathway. The node label color indicates how high the gene was ranked in the differential gene expression analysis, going from black to red with increasing significance. Thickness of an edge reflects the evidence strength for an interaction as reported by the STRING database

**Fig. 9:**
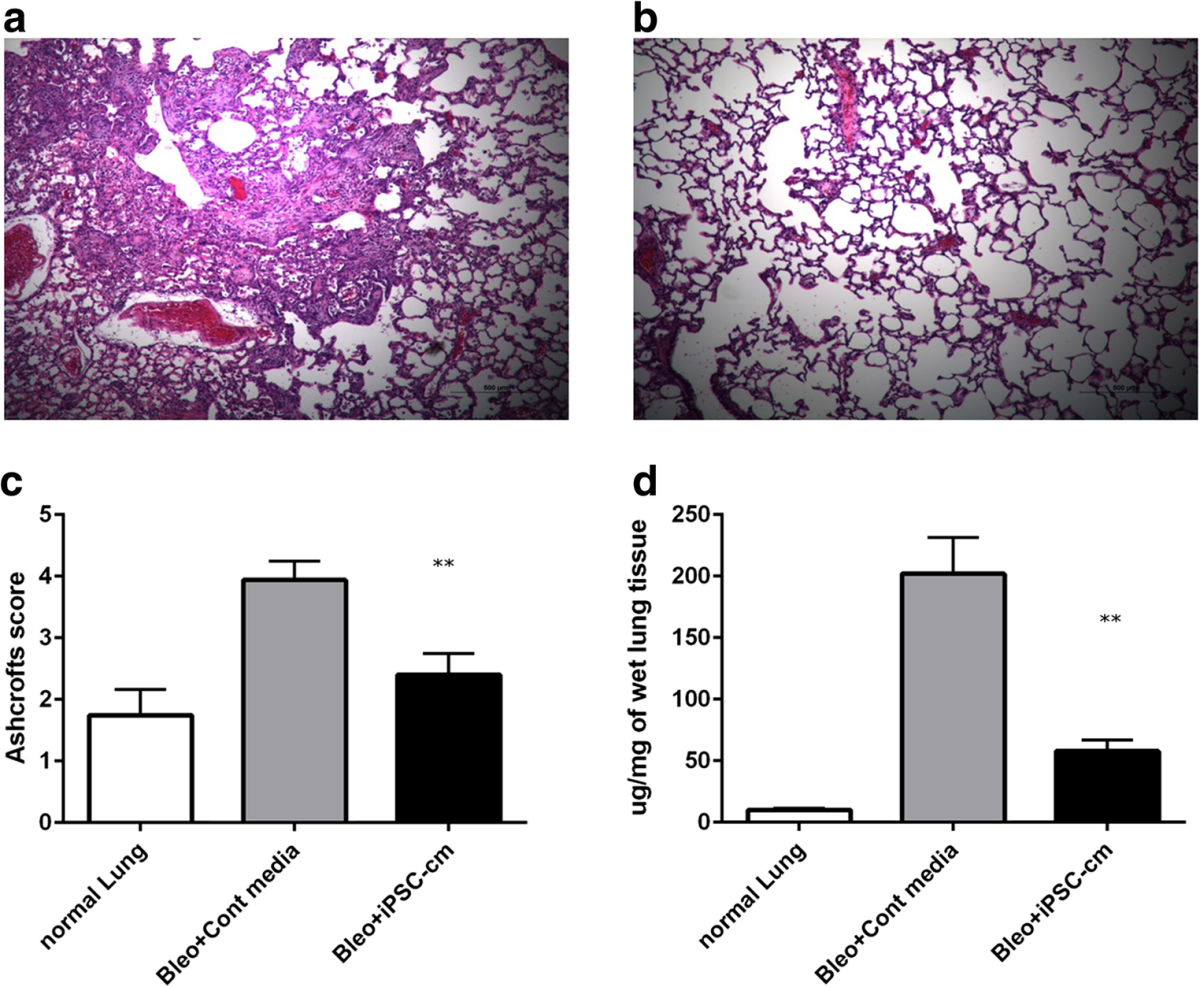
Haematoxylin and eosin staining for lungs treated with control media (**a**) and iPSC conditioned media (**b**). Administration of iPSC-cm to bleomycin-injured rat lungs showed significant attenuation of fibrosis as assessed by semi-quantitative analysis using the Ashcroft score (control media 3.93 ± 0.30 vs iPSC-cm treated 2.4 ± 0.34), healthy controls: (1.74 ± 0.42) (**c**). In accordance, marked reduction in the soluble collagen content was detected in the lungs of iPSC-cm-treated animals compared with bleomycin animals (57.74 ± 9.3 μg/mg vs. 202 ± 29.57 μg/mg). Collagen content of healthy lung was 10.04 ± 1.49 μg/mg (**d**)

### iPSC Cells Maintain Pluripotency After Growing for 24 Hours in Starved Conditions.

**Fig. 10:**
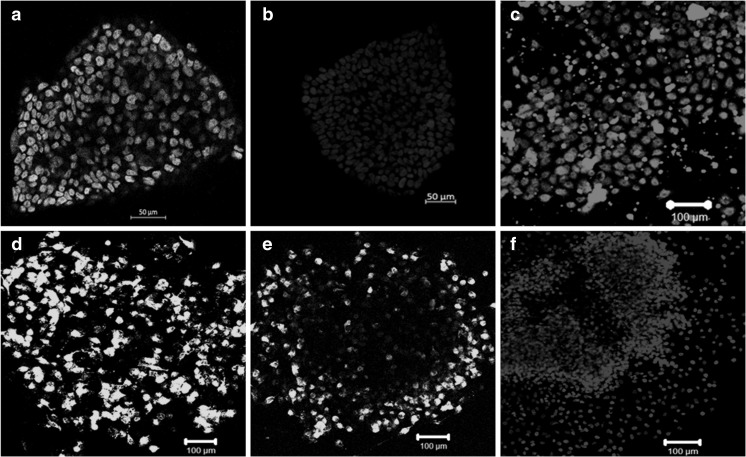
For collection of iPSC-cm the iPSC were grown in media depleted with serum and bFGF for 24 h to conform their pluripotency iPSC colonies were stained with pluripotency markers. **a** Oct3/4 (**b**) Nanog (**c**) Sox-2 (**d**) TRA-1-60 (**e**) TRA-1-81 (f) DAPI

The iPSC colonies were grown in media deprived of serum replacment and bFGF for collection of the secretome.These colonies stained positive for markers of pluripotency, in particular (Fig. 10a) Oct3/4 (Fig. 10b) Nanog (Fig. 10c) Sox-2 (Fig. 10d) TRA-1-60 (Fig. 10e) TRA-1-81 (Fig. 10 f) DAPI.

### iPSC-cm Treatment Reduces Fibrosis in Bleomycin Injured Rat Lung

Bleomycin injured rats when treated with control media showed increased fibrosis (Fig. 9a), in contrast the animals treated with iPSC-cm showed significant improvement in lung architecture as shown by histology (Fig. 9b). Semiquantitative analysis of the histology slides by Ashcrofts score confirmed the observation (control media 3.93 ± 0.30 vs iPSC-cm treated 2.4 ± 0.34) compared to healthy controls: (1.74 ± 0.42) (Fig. 9c). Accordingly the level of soluble collagen as measured by Sircol assay were also reduced in the lungs of iPSC-cm-treated animals compared with bleomycin animals (57.74 ± 9.3 μg/mg vs. 202 ± 29.57 μg/mg). Collagen content of healthy lung was 10.04 ± 1.49 μg/mg (Fig. 9d).

## Discussion

Intratracheal instillation of the iPSC-cm in the bleomycin injured rat lungs reduced fibrosis and total number of macrophages. Additionally, the percentage of M1 and M2 macrophages in the lung also decreased. Gene set enrichment analysis on the microarray data showed the involvement of three main groups of pathways: (a) branching morphogenesis, (b) immune regulation, and (c) tissue regeneration after injury.

Various cell types are involved in regulation of tissue repair after injury, however macrophages play critical role in all stages of repair and fibrosis by displaying remarkable flexibility for adaptation to specific stimuli [37]. The classification of macrophage subpopulations has been revised recently depending on their secretory and gene regulatory pattern [9]. However, for ease of explanation in the current study we used the M1(CD86+) and M2 (CD206+/CD163+) classification based on the surface marker expression [38]. In line with our previous report, significant reduction in collagen content and improved histology was observed seven days after iPSC-cm treatment in bleomycin injured rat lungs ^16^. It is suggested that M1 macrophages are pro-inflammatory and have an antifibrotic effect [39, 40], while M2 macrophages are profibrotic and play a critical role in remodeling and fibrotic process [41].

We observed a reduction in the total interstitial macrophages after iPSC-cm treatment compared to untreated or animals treated with control media. More interestingly, the percentage of both M1 and M2 macrophages was significantly reduced at day 7 after iPSC-cm treatment. A switch between M1 and M2 is suggested to be essential for resolution of inflammation, remodeling and repair and is regulated by various factors [41]. We, however, did not observe a shift in balance between the two macrophage subtypes at day 7 after treatment in our model. To further study possible phenotype transition in time dependent manner, we analyzed the effect of iPSC-cm at different time points in bleomycin injured rat lungs. We did observe an increase in M1 cells at 48 h and 72 h after iPSC-cm treatment, which reduced at day 7. In contrast, the percentage of M2 cells showed a surge at 24 h and then a steady decline over time, suggesting an initial role of M1 macrophages of possibly initiating the repair process by stimulating inflammatory process and that of M2 macrophages of starting initial remodeling.

Additionally, we observed a small population of cells which stained positive for markers of both M1 and M2 (CD68 + CD206 + CD86+) in bleomycin injured lungs. iPSC-cm treatment also reduced the percentage of this cell population significantly. This cell population was not detected in normal lung tissue. The exact role of this population and its possible transition could not be elucidated due to the small number of cells. Their expansion in vitro was not performed since this procedure could alter the cell characteristics and may therefore not represent the exact in vivo physiology. We speculate that these might be the cells in transition from M2 to M1, as M1 are suggested to be antifibrotic [42].

To further evaluate the effect of iPSC-cm on global gene expression, interstitial macrophages were sorted from the lung and transcriptome analysis using gene microarrays was performed. As mentioned before, since the role of interstitial macrophages (IMs) is not fully known [12], we focused our study on IMs. Transcriptome analysis of the isolated IMs revealed very interesting results. Three unique pathways were highlighted after gene set enrichment analysis was performed by comparing the gene expression of macrophages from bleomycin injured lung treated with control media and those treated with the secretome of iPSC. The three pathways involved are (a) morphogenesis of a branching structure (b) toxoplasmosis (immune regulation) and (c) regulation of canonical WNT pathways.

The GO term “Morphogenesis of branching structure” was the most significant pathway affected. The most striking finding was the downregulation of vascular endothelial growth factor A (VEGF-A). VEGF-A is an essential regulator of angiogenesis and has been implicated in pathogenesis of lung fibrosis, and is demonstrated to be a marker of disease severity and progression [43]. More interestingly, anti VEGF treatment attenuated lung fibrosis in experimental studies [44] and also in clinical settings [45]. Recently, a small molecule inhibitor targeting the receptor kinases (Nintedanib) has been approved and is now accepted in clinical application as treatment for IPF [46]. Macrophages produce VEGF to support angiogenesis in hypoxic condition that leads to progression of fibrosis [47]. We observe significant down regulation of VEGF by macrophages in response to iPSC-cm treatment, suggesting that the secretome is targeting a very essential profibrotic pathway thus exerting antifibrotic action.

Further insight on the pathways affected by the secretome of iPSC revealed MHC class II activation. MHC class II are a set of surface proteins that are essential to recognize external pathogens, play an immunoregulatory role and are exclusively present on the antigen presenting cells. A consistent finding in fibrotic lungs is the presence of T cells. Indeed, complex interplay between T effector and T regulatory cells in lung fibrosis has been observed [48]. In patients with IPF both CD4^+^ and CD8^+^ positive cells have been reported, with increased CD8^+^ positive cells indicating towards worse clinical condition [49]. Two broad mechanisms by which T cells might influence the fibrotic process include production of Th-1 or Th-2 cytokines [50, 51] and cell surface molecule interactions with epithelial or mesenchymal cells [52, 53]. In the current study, we observed upregulation of MHC class II genes that negatively regulate T cells [54], possibly representing another antifibrotic mechanism of the iPSC secretome.

Furthermore, the WNT pathway was also influenced by the iPSC secretome. Network analysis revealed upregulation of Axin2. Axin2/Conductin is a negative regulator of WNT signalling pathway [55]. WNT signalling family proteins are essential for lung development and morphogenesis [56] and are linked to pathogenesis of pulmonary fibrosis [57]. Detailed microarray screenings have revealed upregulation of WNT genes in fibrotic lungs [58]. Interestingly, inhibition of WNT inducible signalling proteins reduce lung fibrosis in an experimental mouse model [59]. We observed upregulation of Axin2, a negative regulator of WNT signalling, indicating that the iPSC secretome downregulates WNT signalling and possibly reducing TGFb1 via this mechanisms as reported in our previous study [16]. Another study by Cosin-Roger J et al. [36] demonstrated that M2 macrophages activated WNT signalling in relation to Ulcerative colitis. Therefore, a WNT downregulation may further explain why the iPSC secretome has a beneficial effect on fibrosis by acting through macrophages.

In this study, we report the antifibrotic effect of iPSC secretome in a rat lung injury model being mediated by altering macrophage gene expression. Based on our findings, we propose macrophages as potentially important therapeutic targets, since one subpopulation of macrophages is involved in the pathogenesis and progression of fibrosis, whereas another subpopulation of the macrophages is able to resolve fibrosis. Therefore, a therapeutic approach that tilts the balance towards resolving macrophages would be desirable.

To investigate this hypothesis, we performed an unbiased analysis by applying GSEA on an expression microarray dataset to get a general idea of the effects of iPSC secretome on the IMs gene expression in bleomycin injured rat lung. A limitation of using a rat model is that it is difficult to get a precise mechanistic insight into which human iPSC proteins are binding or affecting which rat macrophage proteins and genes. Indeed, none of the currently publicly available protein interaction databases contains an extensive list of protein–protein interactions between proteins from these two species. We deliberately did not attempt to first map the iPSC proteins to their rat orthologs, as it has been shown repeatedly that gene signaling and regulatory networks are highly divergent between mammalian species, even though the sequences of the proteins involved might be relatively well conserved [60]. Another limitation of our study is that we did not evaluate the effect of iPSC-cm at very early time points i.e. before 24 h after instillation.

Despite these limitations, our pathway analysis does identify several candidate pathways affected by treatment with iPSC medium. Performing in vitro studies on the cells obtained from humans might supplement our findings. Our data indicates that antifibrotic role of iPSC secretome in part is due to its effect on interstitial macrophage in bleomycin injured rat lung. This is the first of its kind of study utilizing network analysis to identify global changes in macrophage genome under influence of iPSC secreteome. More detailed analysis is however required to understand the precise mechanisms of iPSC secretome to further consolidate its use as a potential antifibrotic agent.

## Electronic supplementary material

Below is the link to the electronic supplementary material.

ESM 1 (TIF 600 KB)
