# Supplementary figures and images for: Gene Network Analysis of Interstitial Macrophages After Treatment with Induced Pluripotent Stem Cells Secretome (iPSC-cm) in the Bleomycin Injured Rat Lung
Source: Stem Cell Rev. 2017 Dec 18;14(3):412–24. doi: 10.1007/s12015-017-9790-9 (PMC5960485; doi:10.1007/s12015-017-9790-9)

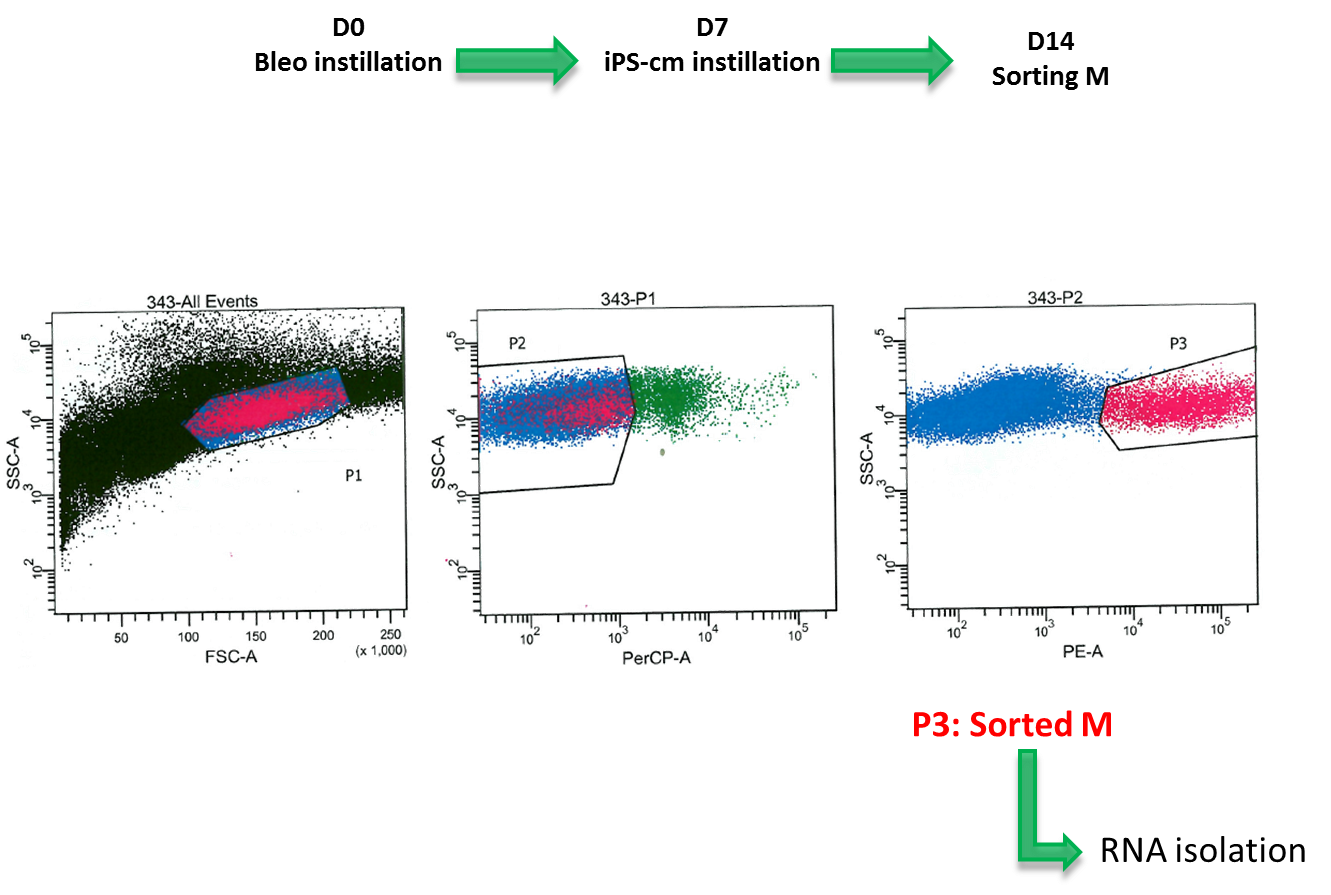

Supplement: Supplementary file 1 — ESM 1 (TIF 600 KB) [file 12015_2017_9790_MOESM1_ESM.tif]
